# Lexical-perceptual integration influences sensorimotor adaptation in speech

**DOI:** 10.3389/fnhum.2014.00208

**Published:** 2014-04-17

**Authors:** Nicolas J. Bourguignon, Shari R. Baum, Douglas M. Shiller

**Affiliations:** ^1^Research Center, Sainte-Justine Hospital, Université de MontréalMontreal, QC, Canada; ^2^School of Speech Pathology and Audiology, Université de MontréalMontreal, QC, Canada; ^3^Centre for Research on Brain, Language and Music, McGill UniversityMontreal, QC, Canada; ^4^School of Communication Sciences and Disorders, McGill UniversityMontreal, QC, Canada

**Keywords:** speech production, sensorimotor integration, lexical effect, altered auditory feedback, language processing

## Abstract

A combination of lexical bias and altered auditory feedback was used to investigate the influence of higher-order linguistic knowledge on the perceptual aspects of speech motor control. Subjects produced monosyllabic real words or pseudo-words containing the vowel [ε] (as in “head”) under conditions of altered auditory feedback involving a decrease in vowel first formant (F1) frequency. This manipulation had the effect of making the vowel sound more similar to [I] (as in “hid”), affecting the lexical status of produced words in two *Lexical-Change* (LC) groups (either changing them from real words to pseudo-words: e.g., *less*—*liss*, or pseudo-words to real words: e.g., *kess*—*kiss*). Two *Non-Lexical-Change* (NLC) control groups underwent the same auditory feedback manipulation during the production of [ε] real- or pseudo-words, only without any resulting change in lexical status (real words to real words: e.g., *mess*—*miss*, or pseudo-words to pseudo-words: e.g., *ness*—*niss*). The results from the LC groups indicate that auditory-feedback-based speech motor learning is sensitive to the lexical status of the stimuli being produced, in that speakers tend to keep their acoustic speech outcomes within the auditory-perceptual space corresponding to the task-related side of the word/non-word boundary (real words or pseudo-words). For the NLC groups, however, no such effect of lexical status is observed.

## Introduction

Linguistic processing of the speech acoustic signal is a notoriously challenging phenomenon: speakers and listeners must selectively navigate ambiguous auditory scenes in real time and filter out irrelevant noise from the ambient environment (Zion Golumbic et al., 2012). To achieve this, the nervous system must be able to extract and integrate various types of perceptual, motor and linguistic information from the incoming speech stream. Instances of information integration across the perceptual and motor domains have been particularly well documented in both speech perception and production (see below), but systematic attempts to understand how these various components interact with each other as well as with more abstract levels of linguistic representation have just begun to take shape (Hickok et al., 2011; Hickok, 2012). The present study aims to contribute to this effort by exploring the possible top-down influence of linguistic information—in this case, the lexical status of words—on the sensorimotor networks supporting spoken language production.

**Figure 1:**
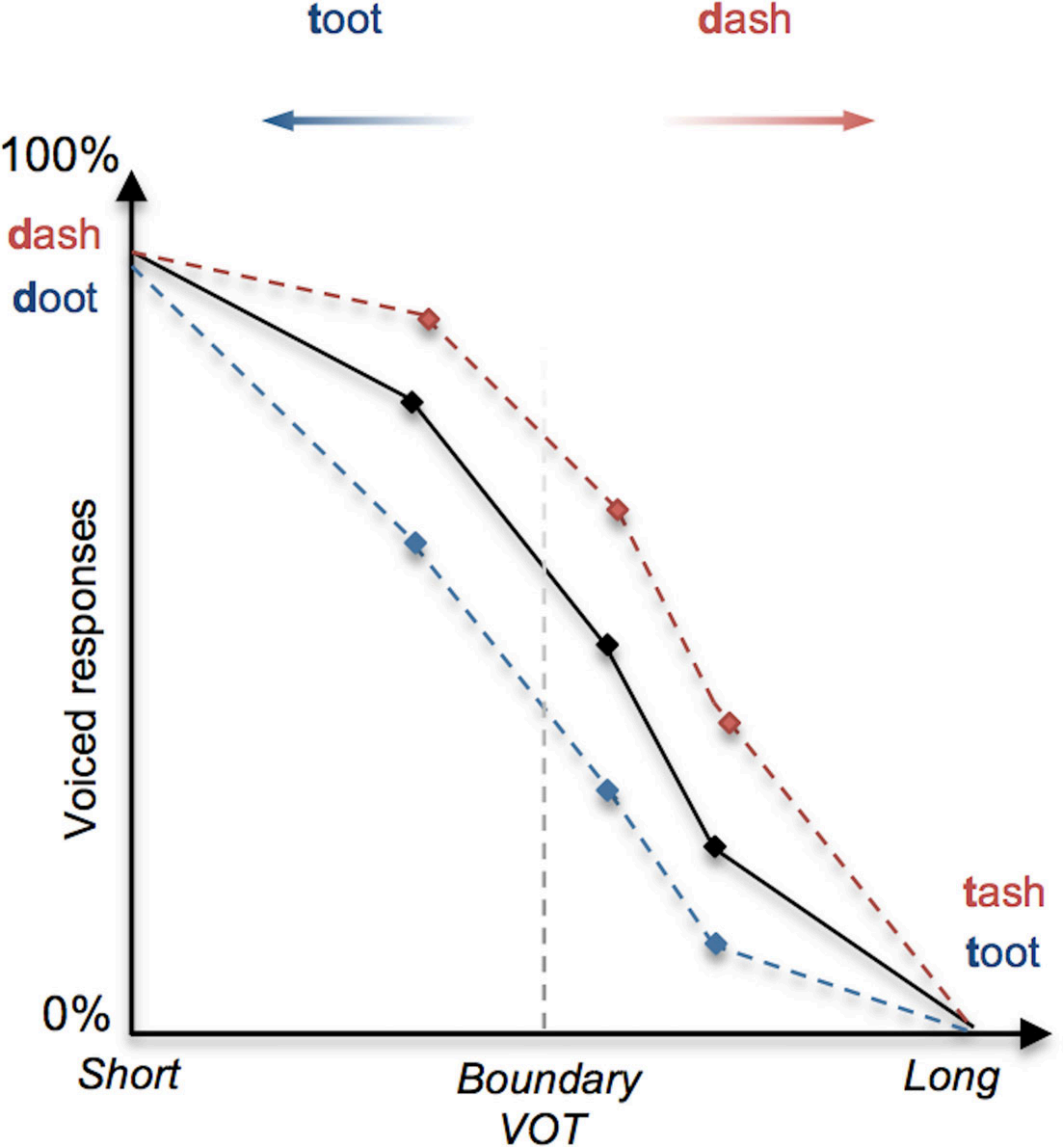
**Illustration of the lexical effect (adapted from Ganong, 1980)**. The perception of a stop consonant as voiced (e.g., [d]) or unvoiced (e.g., [t]) depends primarily upon the duration of the interval between the stop release “burst” and the onset of vocalization (*voice-onset time*, or VOT), whereby a short VOT is perceived as voiced and a long VOT is perceived as unvoiced. When a continuum of stimuli is presented to subjects that involve stop consonants with systematically increasing VOT, the perception shifts from voiced ([d]) to unvoiced ([t]); shown as a decreasing tendency to identify stimuli as voiced. In this example of the *lexical effect*, the proportion of perceived voiced responses (represented on the *y*-axis) for a given VOT stimulus tends to increase (red dashed line) or decrease (blue dashed line) as a function of whether the stimuli correspond to real words (e.g., “dash” and “toot”) or pseudo-words (e.g., “tash” and “doot”).

In language perception, it has long been demonstrated that linguistic context can determine how speech sounds or words are interpreted (Miller et al., 1951; Warren and Sherman, 1974; Ganong, 1980). Amongst the most illustrative examples of this is Ganong's (1980) *lexical effect* on phoneme identification, whereby subjects are presented with a phonetically ambiguous consonant (e.g., between a [d] and a [t] as determined by voice onset time), occurring in a lexical context (e.g., “-ash”) such that one interpretation is consistent with a real-word (e.g., “dash”) and the other maps onto a pseudo-word (“tash”). Ganong's (1980) study and later replications indicate that the identification of a target sound is biased toward real words (Figure 1), effectively shifting phonemic boundaries in favor of existing lexical entries (Connine and Clifton, 1987; Burton et al., 1989; Pitt, 1995).

**Figure 2:**
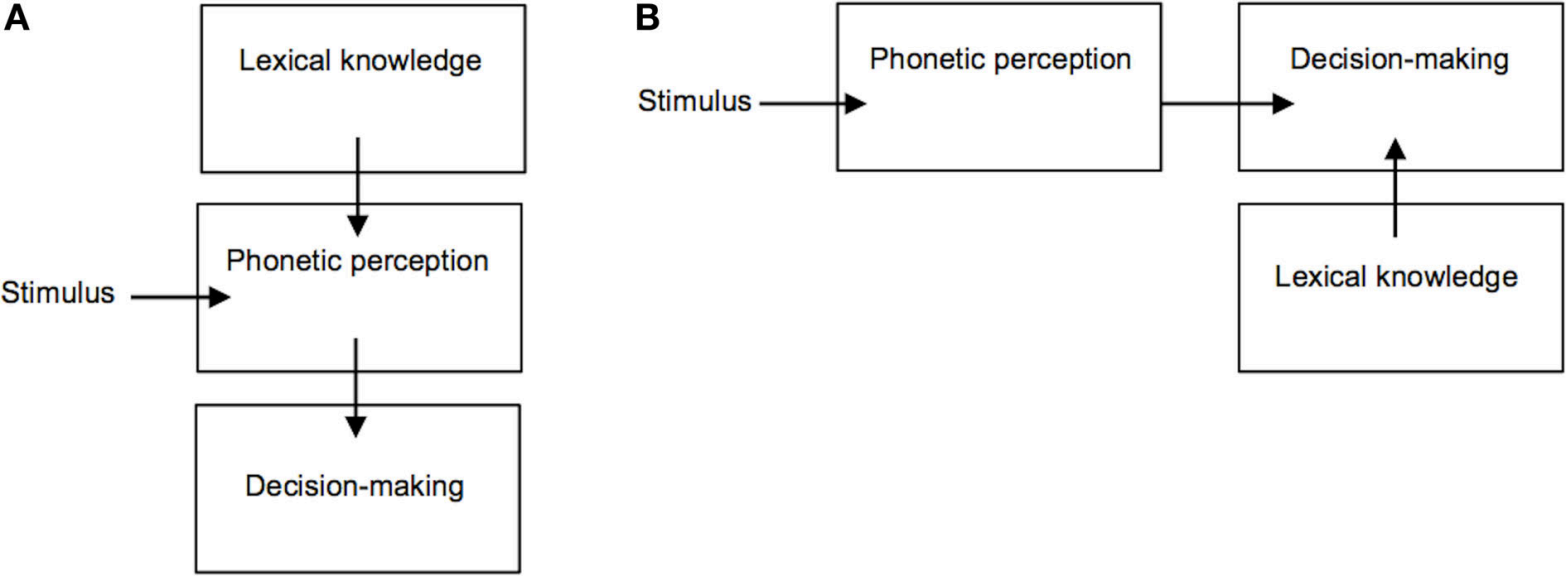
**Competing accounts of the lexical effect on phoneme perception. (A)** Lexical knowledge directly influences phonetic perception; **(B)** Lexical knowledge is used at a later stage for the purpose of explicit decision-making.

These findings have been taken to suggest a direct influence of lexical knowledge on the classification of phonetic categories, however the way in which phonetic-lexical integration takes place and at which stage of the recognition process remains a matter of debate (Fox, 1984; Connine and Clifton, 1987; Pitt and Samuel, 1993; Pitt, 1995; Myers and Blumstein, 2008). One key question is whether this lexical effect emerges from a direct influence of lexical entries on the perception of phonetic input (Figure 2A), or whether lexical knowledge is used at a later stage for the purpose of explicit perceptual decision-making (Figure 2B). Methodologically, disentangling this issue has been complicated by the fact that the lexical effect has been investigated through observation of participants' explicit classification of auditory stimuli (i.e., phoneme identification tasks). Such overt classificatory tasks are not typical of speech perception under naturally occurring conditions, hence it remains unclear to what extent lexical knowledge plays a role in everyday phonetic processing (Fox, 1984). If the lexical effect is primarily related to overt decision-making ability, and not to the perception of speech acoustic properties *per se*, then doing away with any such explicit classificatory task would be expected to reduce or eliminate the influence of lexical knowledge on speech perception. Recent appeal to neuroimaging methods such as fMRI, MEG, and/or EEG (Gow et al., 2008; Myers and Blumstein, 2008) has helped to address this question. In particular, Gow et al. (2008) were able to gain spatially and temporally fine-grained neurophysiological evidence supporting a causal influence of brain areas involved in lexical knowledge on those supporting first-order phonetic processing of ambiguous speech stimuli (similar to those used by Ganong, 1980) *before* areas shown to be involved in perceptual decision-making were recruited (e.g., the left inferior frontal gyrus, see Burton et al., 2000). These findings thus support the notion of a direct top-down influence of lexical representations on speech perception.

Efficient speech production is also known to result from the integration of articulatory motor control with both perceptual and linguistic information, but the influence of these cues upon speech motor processes has so far been investigated using largely non-overlapping approaches (Hickok, 2012 see also Hickok et al., 2011). On the one hand, numerous studies have emphasized that acoustic-phonetic goals guide articulatory control (Houde and Jordan, 1998; Tourville et al., 2008; Shiller et al., 2009). A standard way to demonstrate this influence is to perturb participants' auditory feedback as they produce syllables or words (e.g., “head”) in such a way that a change in acoustic properties (e.g., a decrease in the vowel first formant frequency, or F1) results in the perception of a different speech sound (e.g., a vowel closer to [I], as in “hid”). The perceived deviation of the produced speech acoustic signal from the acoustic target provokes a compensatory change in participants' speech output (e.g., an F1 *increase*), indicating a reliance on the phonetic processing of auditory feedback in guiding and adapting future productions (Houde and Jordan, 1998; Shiller et al., 2009).

On the other hand, psycholinguistic research has pointed to the influence of abstract lexical information on speakers' articulatory patterns (Levelt, 1983, 1989), in particular their tendency to substitute phonemes more often in words or word strings when these substitutions yield real words (*barn door*—*darn bore*) as opposed to pseudo-words (*dart board*—^*^bart doard, Baars et al., 1975; Costa et al., 2006; Oppenheim and Dell, 2008). Interestingly, this so-called *lexical bias* echoes Ganong's lexical effect in showing that the speech production system is biased toward real words relative to pseudo-words.

**Figure 3:**
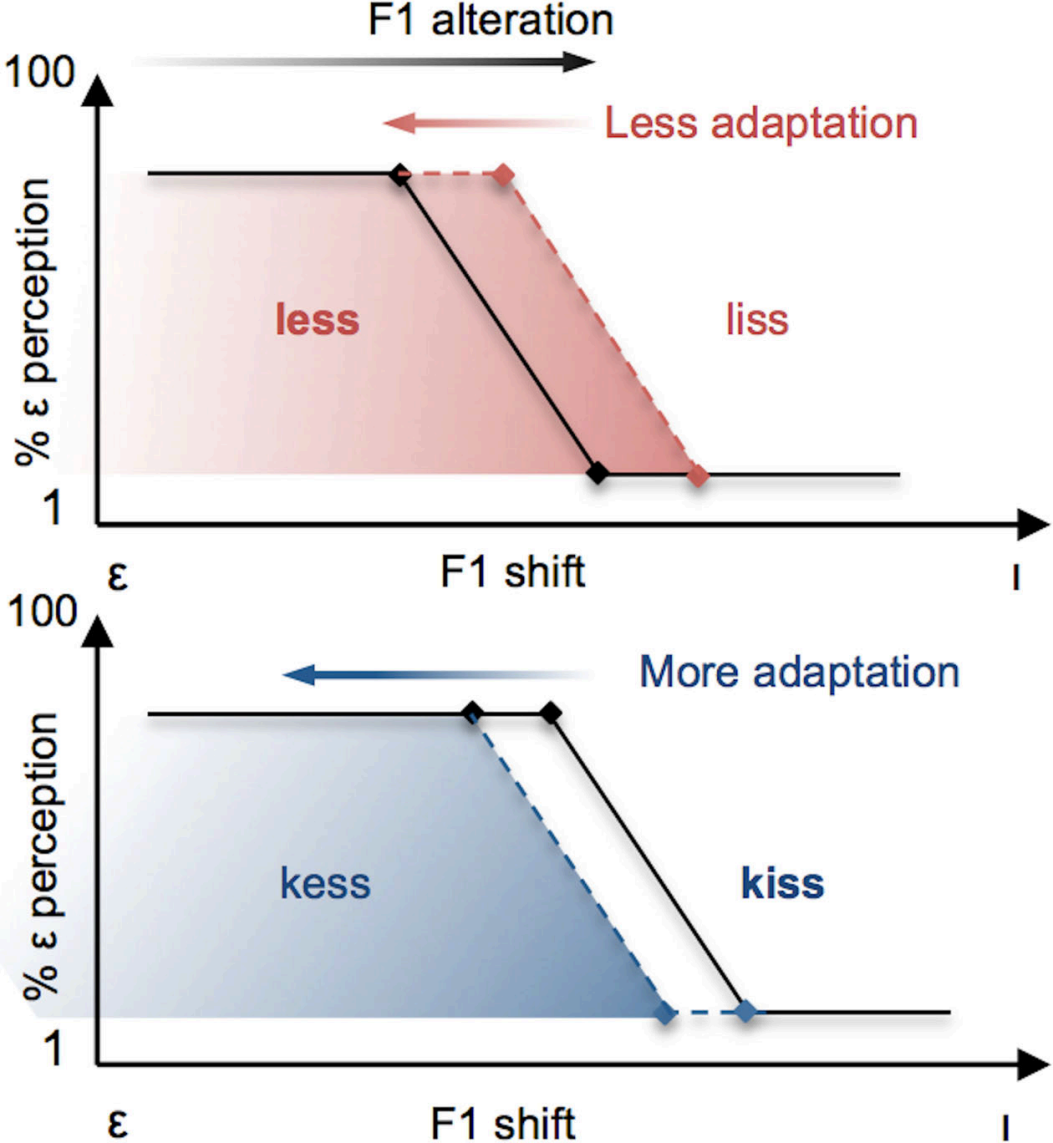
**Schematic illustration of the effects predicted in the present study**. Vowels are altered by a change in F1 frequency, resulting in [ε] productions being perceived more like [ı] (black arrow at top). The resulting compensatory change in speech output (red and blue arrows) has the effect of restoring the subject's perception of their vowel to the original vowel category [ε]. **Top Panel**: F1 vowel alterations that have the effect of changing the lexical status of a real word (e.g., *less*) to a pseudo-word (e.g., *liss*) are predicted to yield reduced compensatory changes in participants' productions (red arrow) as a result of the perceptual lexical effect, whereby the boundary for the vowel contrast is shifted in the direction of [ı] (red dashed line). **Bottom panel**: When the same F1 vowel alteration has the effect of changing the lexical status of a pseudo-word to a real-word, a *larger* compensatory change is expected (blue arrow) due to the perceptual boundary shifting in the direction of the vowel [ε] (blue dashed line).

It is evident from these separate demonstrations that lexical status influences the perception of speech sounds, and that both auditory-perceptual and lexical information play a role in the neural control of speech production. What remains unknown is the extent to which lexical and phonetic information may *interact* during the control of speech production. We investigated this question by combining a variant of Ganong's (1980) lexical manipulation with a paradigm of sensorimotor adaptation to altered auditory feedback during speech production. Specifically, we compared participants' motor adaptation to a perceived decrease in F1 frequency during production of the vowel [ε] (resulting in a vowel perceived to be closer to [I]) under two different conditions. In the *Lexical-Change* (LC) condition, participants produced a series of [ε] real-words (e.g., *less*) or pseudo-words (e.g., *kess*) that were chosen in such a way that the feedback alteration resulted in the perception of stimuli as having a different lexical status (i.e., a real-word perceived as a pseudo-word, or a pseudo-word perceived as a real-word). On the basis of previous demonstrations of the lexical effect (Baars et al., 1975; Ganong, 1980), we hypothesized that in participants' phonetic perception of their own speech auditory feedback, the phonetic boundary would be biased in accordance with lexical status (with the boundary shifting in the direction of the vowel in the non-lexical item, thus enlarging the area along the continuum containing the real-word) and that this bias would be reflected in their patterns of articulatory compensation (see Figure 3). An interaction between phonetic boundaries and the degree of auditory-feedback-based speech compensation has previously been demonstrated in a recent study by Niziolek and Guenther (2013), who observed that the magnitude of compensation to real-time formant alterations was larger when perturbations “push” the perceived sound into the region of the boundary between two phonemes (e.g., between [ε] and [æ]). While that study did not involve manipulations of lexical status or changes to the phonetic boundary itself (capitalizing, rather, on naturally occurring variation in formant frequencies among repeated productions of the same vowel), it supports the notion that the sensory error signal that drives speech motor compensation is defined, in whole or in part, by the proximity of acoustic output to the phonetic boundary.

The speech adaptation effects obtained in the LC condition are compared to a *Non-Lexical-Change* (NLC) control condition, in which participants once again produced either [ε] real words or pseudo-words. However, contrary to the LC condition, the stimuli in this condition were selected such that the F1 shifts did *not* result in a change in lexical status (i.e., a pseudo-word remained a pseudo-word, and a real-word remained a real-word). We expected that subjects in the NLC condition would not exhibit a difference in speech adaptation depending on whether they produced real words or pseudo-words, owing to the lack of any perceptual boundary shift and/or change in lexical status.

## Materials and methods

### Subjects

Forty adult subjects were tested (age range: 18–30 years). All were native speakers of English and had no reported history of speech, language or hearing disorder. Hearing status was verified immediately prior to testing using a pure-tone hearing screening (threshold <20 dB HL at octave frequencies between 250 and 4000 Hz). Subjects provided written informed consent prior to testing. All procedures were approved by the Institutional Review Board, Faculty of Medicine, McGill.

### Stimulus words and group assignment

Subjects were randomly assigned to one of four groups (10 subjects in each, 5 males and 5 females), each of which underwent an identical series of speech production tasks (see Table 1 and *Procedures* below), including the production of monosyllabic words (real-words or pseudo-words) under normal-feedback conditions (*baseline* task) followed by the production of words under conditions of altered auditory feedback (*speech adaptation* task). For all four groups, the words produced in the speech adaptation task contained the vowel [ε] (e.g., “bed” or “geck”). During this task, an acoustic manipulation was carried out in real time such that the vowel [ε] was perceived to be closer to the vowel [I] (as in “bid”; see *Real-time alteration of speech* below). The key difference among the four groups relates to the stimuli produced under these conditions: for two of the groups (*lexical-change*), the stimuli were such that the acoustic manipulation of the vowel changed the lexical status of the item: one group (*LC-1: word to pseudo-word*) produced a set of real-words that, when altered, resulted in a set of pseudo-words (e.g., “test” becoming “tist”), and another group (*LC-2: pseudo-word to word*) produced a set of pseudo-words that, when altered, results in a set of real-words (e.g., “ked” becoming “kid”). For the two other groups (*non-lexical change*), the stimuli were such that the acoustic manipulation of the vowel did *not* alter their lexical status: one group (*NLC-1: word to word*) produced a set of real-words that, when altered acoustically, resulted in another set of real-words (e.g., “bed” becoming “bid”), and a second group (*NLC-2: pseudo-word to pseudo-word*) produced a set of pseudo-words that, when altered acoustically, resulted in a different set of pseudo-words (e.g., “geck” becoming “gick”).

**Table 1:** **Distribution of groups as a function of condition**.

**Lexical change**				**No lexical change**			
**LC1 (G1)**		**LC2 (G2)**		**NLC1 (G3)**		**NLC2 (G4)**	
**ε**	**I**	**ε**	**I**	**ε**	**I**	**ε**	**I**
death	dith	weth	with	bet	bit	jex	jix
depth	dipth	het	hit	pet	pit	bep	bip
nest	nist	fet	fit	ten	tin	tret	trit
less	liss	ket	kit	peck	pick	sten	stin
chess	chiss	kess	kiss	pen	pin	geck	gick
chest	chist	steff	stiff	tech	tick	tetch	titch
test	tist	ked	kid	neck	nick	meck	Mick
vest	vist	detch	ditch	mess	miss	ness	Niss
best	bist	steck	stick	gem	gym	vess	Viss
keg	kig	stell	still				

For subjects in the two lexical-change groups (*LC-1* and *LC-2*), the stimulus set consisted of 10 pairs of items (see Table 1, left). For subjects in the two non-lexical-change groups (*NLC-1* and *NLC-2*), the stimulus set consisted of 9 pairs of items (see Table 1 right). The slight difference in the number of stimuli was due to the difficulty in finding words and pseudo-words that met the required phonetic and lexical criteria for the NLC control groups. For the baseline production task, subjects produced each item 5 times each in a fully randomized order, yielding a total of 100 items for the LC groups, and 90 items for the NLC groups.

The sets of words and pseudo-words used in all of the groups (including the target [ε]-words and the [I]-words resulting from the acoustic manipulation) were matched on a number of criteria, including neighborhood density (Pisoni and Tash, 1974), assessing the number of words that are phonologically similar to the target words, and bi-phonemic probability, which represents how frequently the phoneme pairs in the target words occur together in the lexicon (Vitevitch and Luce, 2004; see Table 2). Both of these variables have been shown to influence word production (Munson and Solomon, 2004; Goldrick and Larson, 2008). A Three-Way analyses of variance (ANOVA), including the factors Condition (Lexical change vs. Non-lexical change), Word Type (Word vs. Pseudo-word) and Vowel ([ε] vs. [I]) was carried out separately for these two measures. For neighborhood density, all main and interaction effects were not significant (*p* > 0.05). A significant difference in bi-phonemic probability was found between [ε] and [I] words [*F*_(1, 69)_ = 8.22, *p* < 0.006]: it was significantly higher in [I]-words relative to [ε]-words (0.006 vs. 0.003). No significant interactions were obtained (all *p*'s > 0.06). No reliable difference was observed for this variable between the different lexical change conditions or word-types (*p* > 0.05), however, thus the four groups remained matched.

**Table 2:** **Control measures for experimental stimuli, including neighborhood density (ND) and word-average bi-phonemic probability (PB)**.

**Condition**	**Type**	**Vowel**	**Example**	**Mean ND (*SD*)**	**Mean PB (*SD*)**
Lexical Change	Pseudo-words	[ε]	*kess*	22.7 (10.5)	0.003 (0.003)
		[ı]	*liss*	22.1 (8.79)	0.007 (0.003)
	Words	[ε]	*less*	21.5 (8.5)	0.004 (0.003)
		[ı]	*kiss*	26.5 (8.5)	0.005 (0.003)
No-Lexical Change	Pseudo-words	[ε]	*bep*	26.4 (9.0)	0.005 (0.004)
		[ı]	*bip*	26.4 (9.0)	0.005 (0.004)
	Words	[ε]	*bed*	21.9 (8.9)	0.004 (0.002)
		[ı]	*bid*	22.7 (7.8)	0.007 (0.004)

### Testing procedures

Speech was recorded in a quiet testing room using a head-mounted microphone (C520, AKG, Germany) and digitized at 16-bit/44.1 kHz on a PC using custom software written in Matlab (Mathworks, MA). Auditory speech signals were presented to subjects using circumaural headphones (880 pro, Beyerdynamic, Germany).

All subjects underwent the following sequence of tasks:

1. *Baseline speech production:* The first task involved the production of a set of stimuli under conditions of normal auditory feedback. Each stimulus was presented orthographically on a computer monitor for 1.5 s, followed by a blank screen for 2 s between items. Subjects were instructed to produce each item as soon as it appeared on screen. Prior to beginning, subjects practiced producing each stimulus item once and their pronunciation was corrected, if necessary, by the experimenter (for all items, subjects were told to produce the vowel “e” as in the word “head,” and the vowel “i” as in the word “hid”).
2. *Test of speech motor adaptation:* For subjects in each group, the baseline production task was followed by a test of speech motor adaptation involving 160 productions of words (or pseudo-words) containing the vowel [ε] from the group's target stimulus list. Word order was randomized. Similar to prior studies of speech adaptation to altered auditory feedback (Purcell and Munhall, 2006; Villacorta et al., 2007; Shiller et al., 2009; Rochet-Capellan and Ostry, 2011), subjects underwent a sequence of auditory feedback conditions involving an initial period of normal feedback (30 trials, *null* phase), followed by a period of practice under conditions of altered auditory feedback (100 trials, *hold* phase), and then finally a period under normal feedback once again, to test for the presence of learning after-effects (30 trials, *after-effect* phase). The auditory feedback manipulation corresponded to a 30% decrease in the frequency of the first spectral peak (the first *formant*, or F1), which resulted in a vowel that was perceived to be more like the vowel [I] (see *Real-time alteration of speech* for details).

### Real-time alteration of speech

The alteration of auditory feedback involved a 30% decrease in the first formant (F1) of the vowel acoustic signal (average shift: 216.3 Hz). The F1 manipulation was carried out using a system that has been described previously (Rochet-Capellan and Ostry, 2011; Shum et al., 2011; Lametti et al., 2012; Mollaei et al., 2013). The microphone signal was amplified and split into two channels: one providing an unprocessed signal and the other altered using a digital signal processor (DSP) to decrease the frequency of all vowel formants (VoiceOne, TC Helicon). The VoiceOne is a commercial DSP designed to alter human speech signals, using source-filter modeling and re-synthesis to independently manipulate fundamental frequency (F0) and formant frequencies (with all formant frequencies shifted proportionally). While the specific processing algorithm is proprietary, the magnitude of formant manipulations and independence of formant and F0 changes was verified empirically by analyzing input and output signals. The vowel alteration was restricted to F1 by splitting both signals into non-overlapping low- and high-frequency components (Wavetek 753 low/high pass filter), and then mixing the low-frequency portion of the processed signal with the high-frequency portion of the unprocessed signal. The filter cutoff used to separate the two signals was set at 1100 Hz for males and 1350 Hz for females, each of which lies roughly half-way between the first and second formant values for the production of the vowel [ε] for men and women respectively (based upon pilot studies). The total signal processing delay was less than 15 ms.

Subjects were encouraged to maintain a constant speaking volume throughout the task through the use of a VU meter presented on the computer display (showing current and peak acoustic signal level during each trial). Subjects were instructed to maintain a target level on the display, which was adjusted at the beginning of the experiment to correspond to a comfortable speaking volume. The subject's perception of his or her own air/bone-conducted speech acoustic signal was reduced by mixing the auditory feedback signal (presented at approximately 75 dB SPL) with speech-shaped masking noise (presented at approximately 60 dB SPL).

### Acoustic analyses

For each word production in the baseline and speech adaptation tasks, a 30 ms segment centered about the midpoint of the vowel was selected using an interactive computer program that displayed the waveform and spectrogram of each utterance, allowing the experimenter to identify a stable, artifact-free region near the vowel center. Mean F1 and F2 (second formant) frequency for each segment was then estimated using LPC analysis in Matlab. LPC parameters were chosen on a per-subject basis in order to minimize the occurrence of clearly spurious formant values. Values of F1 and F2 frequency were used to directly compare vowel acoustic properties during baseline productions of the different stimulus words among the different groups.

Analysis of vowel acoustics during the speech adaptation task was restricted to F1 frequency, as the compensatory response was primarily observed in this acoustic parameter. During the adaptation task, changes in F2 under conditions of altered auditory feedback rarely exceeded 1% of baseline values, consistent with other reports of vowel feedback manipulations that were restricted to F1 (see, e.g., Purcell and Munhall, 2006; Villacorta et al., 2007). Following Villacorta et al. (2007) and others (Rochet-Capellan and Ostry, 2011; Shum et al., 2011; Lametti et al., 2012; Mollaei et al., 2013), changes in vowel production during the speech adaptation task were computed as the proportion change in F1 frequency relative to the mean values during the *null* phase (averaged over trials 11–30). Such normalized units (which convey changes in formant values relative to a nominal value of 1) are preferable to non-normalized units as they account for individual differences in baseline acoustic properties (e.g., between men and women). Differences in speech adaptation between the different groups were evaluated at three time-points: (1) the beginning of the Hold phase (averaged over trials 31–60) under conditions of altered auditory feedback, (2) the end of the Hold phase (trials 101–130) under conditions of altered auditory feedback, and (3) during the After-Effect phase following removal of the feedback manipulation (trials 131–160).

## Results

### Baseline measures

Baseline productions of [ε]-words produced in the four different groups were compared in order to verify that mean F1 and F2 values were similar among the different real-word and pseudo-word conditions (thus yielding similar magnitudes of F1 alteration during the speech adaptation task). Mean and SD values of F1 for the two lexical-change groups (LC-1 and LC-2) and the two non-lexical-change control groups (NLC-1 and NLC-2) respectively were: 756.7(97.7), 670.0(132.4), 722.7(105.1), and 739.3(122.4) Hz. Mean and SD values of F2 for the four groups respectively were: 1887.6(176.9), 1828.2(162.7), 1831.6(150.9), and 1828.9(195.5) Hz. A One-Way ANOVA was carried out to assess any differences among the four conditions in F1 and F2. No significant differences were found between conditions for either formant [F1: *F*_(3, 36)_ = 1.12, *p* = 0.35; F2: *F*_(3, 36)_ = 0.30, *p* = 0.822].

### Speech adaptation

**Figure 4:**
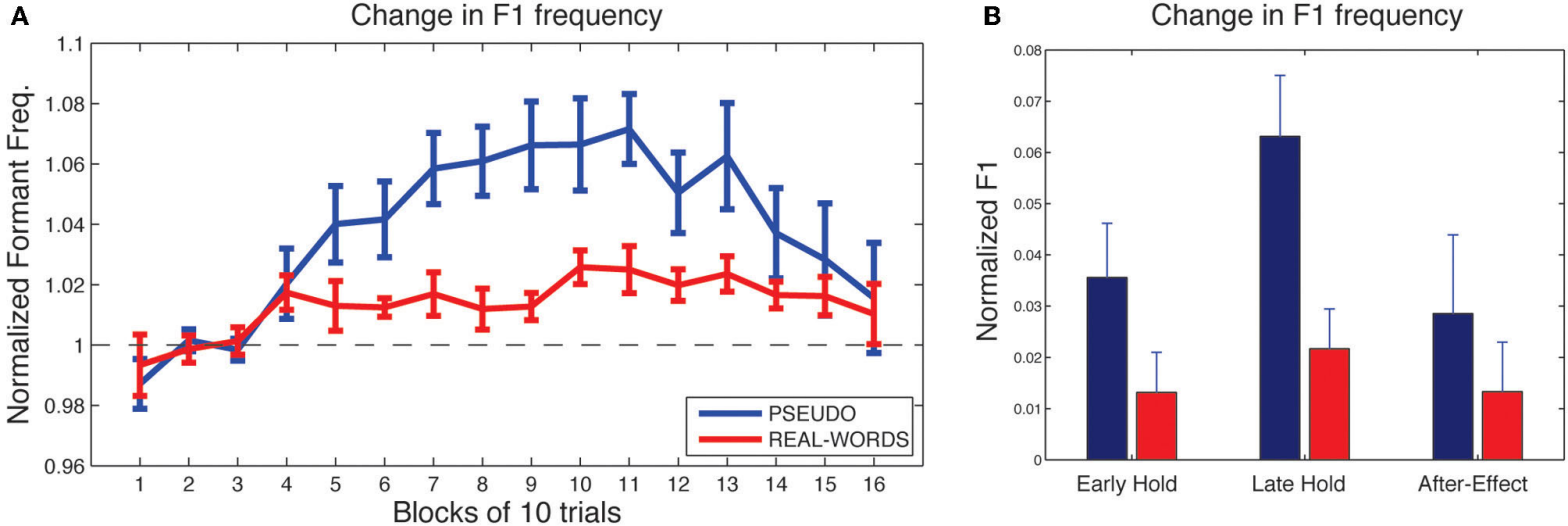
**Results of speech adaptation task for the two lexical-change groups (LC-1 and LC-2). (A)** Mean F1 frequency (normalized units) is shown throughout the task, averaged over successive blocks of 10 trials each. The magnitude of the change in F1 is notably greater for LC-2 (blue line), compared with LC-1 (red line). **(B)**. Mean normalized F1 at three key time-points during the adaptation task.

**Figure 5:**
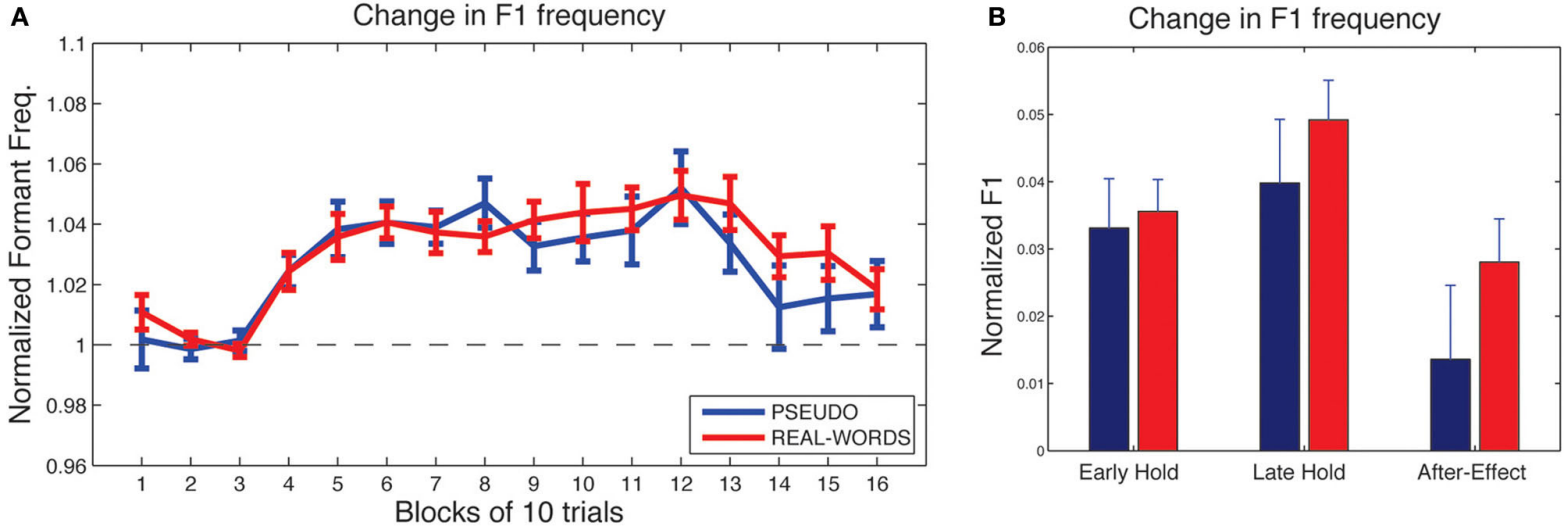
**Results of speech adaptation task for the two non-lexical-change groups (NLC-1 and NLC-2). (A)** Mean normalized F1 frequency is shown throughout the task, averaged over successive blocks of 10 trials each. The magnitude of the change in F1 is comparable for the two groups. **(B)** Mean normalized F1 at three key time-points during the adaptation task.

As shown in Figure 4A, subjects in both lexical-change groups (LC-1, producing real-words, and LC-2, producing pseudo-words) exhibited a change in F1 frequency (compensatory increase) in response to the auditory feedback manipulation, though with a notable difference between groups in the magnitude of the response. The compensatory change in F1 can be seen to build up throughout the *hold* phase and then diminish gradually during the *after-effect* phase. For the two *non-lexical-change* groups (NLC-1, producing real-words, and NLC-2, producing pseudo-words), the time-course of compensatory change in F1 is shown in Figure 5A. For both groups, a robust increase in F1 frequency can be seen following the onset of altered auditory feedback. In contrast with the two LC groups, however, very little difference in the magnitude of compensation is observed throughout the training.

As a first analysis step, we evaluated for each group whether the maximum observed compensatory changes in formant values (at the end of the *hold* phase) were statistically reliable, using Holm-Bonferroni-corrected single-sample *t*-tests comparing formant values against a hypothesized mean of 1 (the value representing no difference from baseline in normalized units). Note that in each of the two lexical-change groups (LC-1 and LC-2), a single subject exhibited a change in F1 frequency in the direction *opposite* that of the compensatory response, as indicated by a statistically reliable F1 *decrease* at the end of the hold phase (trials 101–130) relative to baseline for those two subjects (*p* < 0.01). No subjects in the non-lexical-change groups (NLC-1 and NLC-2) exhibited a reliable F1 change in the negative direction. The presence of subjects who show such “following” responses to feedback perturbations has been noted in previous studies (e.g., Villacorta et al., 2007; MacDonald et al., 2010). In the present study, such subjects were excluded from subsequent analyses to avoid averaging across responses that were qualitatively different (negative vs. positive change). All four groups were found to exhibit a reliable compensatory change in F1 at the end of the hold phase [LC-1: (8) = 2.78, *p* = 0.018; LC-2: *t*_(8)_ = 5.30, *p* < 0.001; NLC-1: *t*_(9)_ = 8.39, *p* < 0.001; NLC-2: *t*_(9)_ = 4.2, *p* = 0.003].

Mean changes in formant values relative to baseline at three time-points during the testing sequence are shown for the lexical-change groups in Figure 4B, and for the non-lexical-change groups in Figure 5B. To compare the magnitude of the adaptation response among the two lexical-change conditions, the two word-production conditions, and the three time-points, an omnibus Three-Way mixed-factorial ANOVA was carried out with WORD (word vs. non-word) as one between-subjects factor, LEXICAL (lexical change vs. non-lexical-change) as a second between-subjects factor, and PHASE (Early-hold, Late-hold, After-effect) as a within-subjects factor. The two between-subject main effects (WORD and LEXICAL) were found to be non-significant [WORD: *F*_(1, 34)_ = 0.87, *p* = 0.36; PHASE: *F*_(1, 34)_ = 0.22, *p* = 0.64], however a reliable 2-way interaction between WORD and LEXICAL was observed [*F*_(1, 34)_ = 4.36, *p* = 0.04]. A highly significant main effect of the within-subject variable PHASE was also observed [*F*_(2, 68)_ = 22.49, *p* < 0.001], but there was no reliable 2-way interaction between PHASE and either of the two group variables [PHASE × WORD: *F*_(2, 68)_ = 2.52, *p* = 0.09 PHASE × LEXICAL: *F*_(2, 68)_ = 1.20, *p* = 0.31]. The 3-way interaction was also found to be non-significant [*F*_(2, 68)_ = 2.046, *p* = 0.14].

The 2-way interaction between WORD and LEXICAL conditions is of particular interest, since our main prediction involves a difference in the degree of adaptation between the word and non-word production under the lexical-change condition, with no such difference predicted between word groups under the non-lexical-change condition. *Post-hoc* pair-wise comparisons were carried out using Holm-Bonferroni-corrected *t*-tests to examine the reliability of these simple group effects. The tests were carried out on adaptation performance at the end of the training period (i.e., the Late-hold phase), as this represented the moment at which speech adaptation was maximal for all groups. A reliable difference in the magnitude of speech adaptation was observed between the groups producing real-words and pseudo-words in the lexical-change condition [*t*_(16)_ = 2.91, *p* = 0.04], while no significant difference was observed between the two word conditions in the non-lexical-change condition [*t*_(18)_ = 0.84, *p* = 0.41]. A reliable difference was also observed between the two groups producing real words, one in the lexical-change condition and one in the non-lexical-change condition [*t*_(17)_ = 2.86, *p* = 0.03]. No such difference was observed between the two groups producing pseudo-words [*t*_(17)_ = 1.55, *p* = 0.28].

## Discussion

This study investigated the possible interaction between perceptual and lexical information in guiding articulatory movements during spoken speech. Our hypothesis was that compensatory changes in speakers' articulatory patterns following an auditory-feedback perturbation affecting vowel quality would reflect sensitivity to the lexical status of the words produced. In particular, we predicted that the degree of compensation would vary when the auditory feedback manipulation had an effect on the lexical status of the word being produced (changing a real word into a pseudo-word, or vice versa).

The present findings support this prediction: articulatory compensation to a decrease in F1 inducing a shift from real words to pseudo-words (group LC-1) was found to be significantly less than when the same F1 perturbations provoked a reverse shift from pseudo-words to real words (LC-2). Furthermore, the speech compensatory response for the LC-1 group (producing real-words under conditions of lexical-change) was found to be significantly less than that observed for the group producing real-words under non-lexical-change conditions (NLC-1). Crucially, no such difference in speech compensation was observed between the groups producing real-words and pseudo-words under control conditions in which the same auditory feedback manipulation induced no lexical change (groups NLC-1 and NLC-2 respectively). The observed difference in adaptation magnitude between the two LC groups can be interpreted as an extension of the original lexical effect on phoneme perception demonstrated by Ganong (1980). More precisely, this finding suggests that the lexical effect on perception can also be found in production, resulting from the fact that speakers tend to keep their acoustic speech outcomes within the auditory-perceptual space corresponding to the task-related side of the word/non-word boundary (real words or pseudo-words; see Figure 3).

The conceptual and methodological payoffs of combining altered auditory feedback and lexical bias into a single experimental paradigm are twofold. First, evidence for lexically driven motor adaptation to auditory perturbations demonstrates for the first time a concurrent influence of phonetic and lexical information on the control of spoken speech, indicating that articulatory plasticity is in part constrained by the structure of abstract lexical knowledge. Second, observing a lexical effect through participants' speech productions bypasses the methodological shortcomings associated with explicit perceptual decision-making tasks, and thus strongly supports the view that the lexical influence on perception involves a change in phonetic processing (e.g., Gow et al., 2008), and is not simply the result of a bias in lexically-driven decision-making.

A number of early studies of top-down or contextual influences on phonetic perception indicated that such effects may emerge only in slower reaction time ranges, suggesting that the effect is post-perceptual and thus supporting an independent, input-driven phoneme perception mechanism, free of higher-level factors (e.g., Fox, 1984; Connine and Clifton, 1987; Cutler et al., 1987; Miller and Dexter, 1988; Burton et al., 1989). While the present study does not allow for a precise characterization of the timing with which lexical knowledge exerts, or stops exerting its influence on speech perceptual or motor functions, the present results are more consistent with the view of a more interactive speech perceptual system, with parallel processing of numerous streams of information, permitting early integration during speech and language processing. This view is also supported by neurobiological evidence for an early interaction between word-form representation areas (e.g., the supramarginal gyrus) and lower-level perceptual cortices (e.g., the superior temporal gyrus) in a time range prior to that of decision-making (Gow et al., 2008).

In current neuro-computational models of the sensorimotor control of speech production, such as the DIVA model (Guenther et al., 2006; Tourville and Guenther, 2011) or the State Feedback Control model of speech motor control (Houde and Nagarajan, 2011), changes in auditory feedback, such as those introduced in the present study, result in a mismatch between actual and expected auditory consequences of speech production. This gives rise to an auditory *error signal* that is used (to varying degrees) to directly alter the control of the oral motor system, as well as to update a predictive feed-forward control mechanism, thus improving subsequent speech motor plans. In the present study, the observed difference in the degree of sensory-motor adaptation between the two LC groups indicates an influence of lexical status on the perception of auditory feedback *prior* to the establishment of the auditory error signal. Recent MEG studies have shown that auditory feedback processing, including the comparison between actual and predicted acoustic outcomes under conditions of altered auditory feedback, occurs at latencies of less than 100 ms (Aliu et al., 2009; Niziolek et al., 2013). There is also behavioral evidence of speech compensatory responses to unexpected auditory feedback perturbations at latencies of less than 250 ms that reflect an influence of the phonetic boundary (Niziolek and Guenther, 2013). Therefore, the present result suggests an influence of lexical status on sensorimotor function at a similarly short latency.

The present findings indicate that the perceptual and motor sub-systems of the speech apparatus interact to a certain extent with higher-order lexical information, although the way in which this interaction takes place and at which stage of the production process remains to be determined. This notion is absent in current neurocognitive models of speech production, such as the DIVA model (Guenther et al., 2006). Hickok (2012) recently proposed a hierarchical psycholinguistic-motor control model of speech, whereby activation of lexical information not only constitutes the starting point toward speech output (in the same vein as earlier models of production, cf. Indefrey and Levelt, 2004), but would also exert a possible influence on the acoustic and somatosensory feedback loops underlying spoken speech. Further work will hopefully yield a more detailed analysis of where and when this interaction takes place. Notice also that lexical-sensorimotor interactions may be present on the somatosensory side of speech production, which would be worth exploring in future studies using real-time somatosensory perturbations during speech (e.g., Tremblay et al., 2008).

### Conflict of interest statement

The authors declare that the research was conducted in the absence of any commercial or financial relationships that could be construed as a potential conflict of interest.

## References

[B1] Aliu S. O. Houde J. F. Nagarajan S. S. (2009). Motor-induced suppression of the auditory cortex. J. Cogn. Neurosci. 21, 791–802 10.1162/jocn.2009.21055 18593265 PMC2944400

[B2] Baars B. J. Motley M. T. McKay D. G. (1975). Output editing for lexical status in artificially elicited slips of the tongue. J. Verb. Learn. Verb. Behav. 14, 382–391 10.1016/S0022-5371(75)80017-X

[B3] Burton M. W. Baum S. R. Blumstein S. E. (1989). Lexical effects on the phonetic categorization of speech: the role of acoustic structure. J. Exp. Psychol. Hum. Percept. Perform. 15, 567–575 10.1037/0096-1523.15.3.567 2527963

[B4] Burton M. W. Small S. L. Blumstein S. E. (2000). The role of segmentation in phonological processing: an fMRI investigation. J. Cogn. Neurosci. 12, 679–690 10.1162/089892900562309 10936919

[B5] Connine C. M. Clifton C. (1987). Interactive use of lexical information in speech perception. J. Exp. Psychol. Hum. Percept. Perform. 13, 291–299 10.1037/0096-1523.13.2.291 2953858

[B6] Costa A. Roelstraete B. Hartsuiker R. J. (2006). The lexical bias effect in bilingual speech production: evidence for feedback between lexical and sublexical levels across languages. Psychon. Bull. Rev. 13, 972–977 10.3758/BF03213911 17484421

[B7] Cutler A. Mehler J. Morris D. Segui J. (1987). Phoneme identification and the lexicon. Cogn. Psychol. 19, 141–177 10.1016/0010-0285(87)90010-7

[B8] Fox R. A. (1984). Effect of lexical status on phonetic categorization. J. Exp. Child Psychol. Hum. Percept. Perform. 10, 526–540 10.1037/0096-1523.10.4.526 6235317

[B9] Ganong W. F. (1980). Phonetic categorization in auditory word perception. J. Exp. Psychol. Hum. Percept. Perform. 6, 110–125 10.1037/0096-1523.6.1.110 6444985

[B10] Goldrick M. Larson M. (2008). Phonotactic probability influences speech production. Cognition 107, 1155–1164 10.1016/j.cognition.2007.11.009 18096149

[B11] Gow D. W. Jr. Segawa J. A. Ahlfors S. P. Lin F.-H. (2008). Lexical influences on speech perception: a Granger causality analysis of MEG and EEG sources estimates. Neuroimage 43, 614–623 10.1016/j.neuroimage.2008.07.027 18703146 PMC2585985

[B12] Guenther F. H. Ghosh S. S. Tourville J. A. (2006). Neural modeling and imaging of the cortical interactions underlying syllable production. Brain Lang. 96, 280–301 10.1016/j.bandl.2005.06.001 16040108 PMC1473986

[B13] Hickok G. (2012). Computational neuroanatomy of speech production. Nat. Rev. Neurosci. 13, 135–145 10.1038/nrn3158 22218206 PMC5367153

[B14] Hickok G. Houde J. F. Rong F. (2011). Sensorimotor integration in speech processing: computational basis and neural organization. Neuron 69, 407–422 10.1016/j.neuron.2011.01.019 21315253 PMC3057382

[B15] Houde J. D. Nagarajan S. S. (2011). Speech production as state feedback control. Front. Hum. Neurosci. 5:82 10.3389/fnhum.2011.00082 22046152 PMC3200525

[B16] Houde J. Jordan M. I. (1998). Sensorimotor adaptation in speech production. Science 279, 1213–1216 10.1126/science.279.5354.1213 9469813

[B17] Indefrey P. Levelt W. J. M. (2004). The spatial and temporal signatures of word production components. Cognition 92, 101–144 10.1016/j.cognition.2002.06.001 15037128

[B18] Lametti D. R. Nasir S. M. Ostry D. J. (2012). Sensory preference in speech production revealed by simultaneous alteration of auditory and somatosensory feedback. J. Neurosci. 32, 9351–9358 10.1523/JNEUROSCI.0404-12.2012 22764242 PMC3404292

[B19] Levelt W. J. M. (1983). Monitoringand self-repair in speech. Cognition 14, 41–104 10.1016/0010-0277(83)90026-4 6685011

[B20] Levelt W. J. M. (1989). Speaking: From Intention to Articulation. Cambridge, MA, MIT Press

[B21] MacDonald E. N. Goldberg R. Munhall K. G. (2010). Compensations in response to real-time formant perturbations of different magnitudes. J. Acoust. Soc. Am. 127, 1059–1068 10.1121/1.3278606 20136227 PMC2830267

[B22] Miller G. A. Heise G. A. Lichten W. (1951). The intelligibility of speech as a function of the context of the test materials. J. Exp. Psychol. 41, 329–335 10.1037/h0062491 14861384

[B23] Miller J. L. Dexter E. R. (1988). Effects of speaking rate and lexical status on phonetic perception. J. Exp. Psychol. Hum. Percept. Perform. 14, 369–378 10.1037/0096-1523.14.3.369 2971767

[B24] Mollaei F. Shiller D. M. Gracco V. L. (2013). Sensorimotor adaptation of speech in Parkinson's disease. Mov. Disord. 28, 1668–1674 10.1002/mds.25588 23861349 PMC3812368

[B25] Munson B. Solomon N. P. (2004). The effect of phonological neighborhood density on vowel articulation. J. Speech Lang. Hear. Res. 47, 1048–1105 10.1044/1092-4388(2004/078) 15605431 PMC4336539

[B26] Myers E. B. Blumstein S. E. (2008). The neural bases of the lexical effect: an fMRI investigation. Cereb. Cortex 18, 278–288 10.1093/cercor/bhm053 17504782

[B27] Niziolek C. A. Guenther F. H. (2013). Vowel category boundaries enhance cortical and behavioral responses to speech feedback alterations. J. Neurosci. 33, 12090–12098 10.1523/JNEUROSCI.1008-13.2013 23864694 PMC3713738

[B28] Niziolek C. A. Nagarajan S. S. Houde J. F. (2013). What does motor efference copy represent? Evidence from speech production. J. Neurosci. 33, 16110–16116 10.1523/JNEUROSCI.2137-13.2013 24107944 PMC3792453

[B29] Oppenheim G. Dell G. S. (2008). Inner speech slips exhibit lexical bias, but not the phonemic similarity effect. Cognition 106, 528–537 10.1016/j.cognition.2007.02.006 17407776 PMC2435259

[B31a] Pisoni D. B. Tash J. (1974). Reaction times to comparisons within and across phonetic categories. Percept. Psychophys. 15, 285–290 10.3758/BF03213946 23226881 PMC3515635

[B30] Pitt M. A. (1995). The locus of the lexical shift in phoneme identification. J. Exp. Psychol. Learn. Mem. Cogn. 21, 1037–1052 10.1037/0278-7393.21.4.1037 7673866

[B31] Pitt M. A. Samuel A. G. (1993). An empirical and meta-analytic evaluation of the phoneme identification task. J. Exp. Psychol. Hum. Percept. Perform. 19, 699–725 10.1037/0096-1523.19.4.699 8409855

[B32] Purcell D. W. Munhall K. G. (2006). Adaptive control of vowel formant frequency: evidence from real-time formant manipulation. J. Acoust. Soc. Am. 120, 966–977 10.1121/1.2217714 16938984

[B33] Rochet-Capellan A. Ostry D. J. (2011). Simultaneous acquisition of multiple auditory–motor transformations in speech. J. Neurosci. 31, 2657–2662 10.1523/JNEUROSCI.6020-10.2011 21325534 PMC3079285

[B34] Shiller D. M. Sato M. Gracco V. L. Baum S. R. (2009). Perceptual recalibration of speech sounds following speech motor learning. J. Acoust. Soc. Am. 125, 1103–1113 10.1121/1.3058638 19206885

[B35] Shum M. Shiller D. M. Baum S. R. Gracco V. L. (2011). Sensorimotor integration for speech motor learning involves the inferior parietal cortex. Eur. J. Neurosci. 34, 1817–1822 10.1111/j.1460-9568.2011.07889.x 22098364 PMC3703318

[B36a] Tourville J. A. Guenther F. H. (2011). The DIVA model: a neural theory of speech acquisition and production. Lang. Cogn. Process. 26, 952–981 10.1080/01690960903498424 23667281 PMC3650855

[B36] Tourville J. A. Reilly K. J. Guenther F. H. (2008). Neural mechanisms underlying auditory feedback control of speech. Neuroimage 39, 1429–1443 10.1016/j.neuroimage.2007.09.054 18035557 PMC3658624

[B37] Tremblay S. Houle G. Ostry D. J. (2008). Specificity of speech motor learning. J. Neurosci. 28, 2426–2434 10.1523/JNEUROSCI.4196-07.2008 18322088 PMC6671181

[B38] Villacorta V. M. Perkell J. S. Guenther F. H. (2007). Sensorimotor adaptation to feedback perturbations of vowel acoustics and its relation to perception. J. Acoust. Soc. Am. 122, 2306–2319 10.1121/1.2773966 17902866

[B39] Vitevitch M. Luce P. A. (2004). A Web-based interface to calculate phonotactic probability for words and nonwords in English. Behav. Res. Methods Instrum. Comput. 36, 481–487 10.3758/BF03195594 15641436 PMC2553700

[B40] Warren R. M. Sherman G. L. (1974). Phonemic restorations based on subsequent context. Percept. Psychophys. 16, 150–156 10.3758/BF03203268

[B41] Zion Golumbic E. M. Poeppel D. Schroeder C. E. (2012). Temporal context in speech processing and attentional stream selection: a behavioral and neural perspective. Brain Lang. 122, 151–161 10.1016/j.bandl.2011.12.010 22285024 PMC3340429

